# Bone Age Assessment Using Various Medical Imaging Techniques Enhanced by Artificial Intelligence

**DOI:** 10.3390/diagnostics15030257

**Published:** 2025-01-23

**Authors:** Wenhao Yuan, Pei Fan, Le Zhang, Wenbiao Pan, Liwei Zhang

**Affiliations:** 1 Information Technology Center, Wenzhou Medical University, Wenzhou 325035, China; ywhpost@wmu.edu.cn (W.Y.);; 2 Department of Mathematics and Statistics, Chonnam National University, Gwangju 61186, Republic of Korea; 3 Department of Orthopaedics, The Second Affiliated Hospital and Yuying Children’s Hospital of Wenzhou Medical University, Wenzhou 325027, China; 4 State-Owned Assets and Laboratory Management Office, Wenzhou University, Wenzhou 325035, China

**Keywords:** bone age assessment, AI, X-ray imaging, MRI, CT, ultrasound imaging

## Abstract

Bone age (BA) reflects skeletal maturity and is crucial in clinical and forensic contexts, particularly for growth assessment, adult height prediction, and managing conditions like short stature and precocious puberty, often using X-ray, MRI, CT, or ultrasound imaging. Traditional BA assessment methods, including the Greulich-Pyle and Tanner–Whitehouse techniques, compare morphological changes to reference atlases. Despite their effectiveness, factors like genetics and environment complicate evaluations, emphasizing the need for new methods that account for comprehensive variations in skeletal maturity. The limitations of classical BA assessment methods increase the demand for automated solutions. The first automated tool, HANDX, was introduced in 1989. Researchers now focus on developing reliable artificial intelligence (AI)-driven tools, utilizing machine learning and deep learning techniques to improve accuracy and efficiency in BA evaluations, addressing traditional methods’ shortcomings. Recent reviews on BA assessment methods rarely compare AI-based approaches across imaging technologies. This article explores advancements in BA estimation, focusing on machine learning methods and their clinical implications while providing a historical context and highlighting each approach’s benefits and limitations.

## 1. Introduction

Bone age (BA) reflects an individual’s skeletal and biological maturity, differing from chronological age (CA) [1]. BA estimation is essential in clinical and forensic (legal) medicine like orthodontics, orthopedics, and pediatric endocrinology [2]. Evaluating the growth and maturation in pediatrics is of paramount importance, especially in the context of various syndromes and endocrine conditions that are linked to short stature, often characterized by a delay in BA development. Additionally, it serves as a tool for forecasting the eventual adult height [3,4,5]. Furthermore, the growing incidence of precocious puberty has heightened the interest in the developmental aspects of children and the implementation of growth hormone therapies, thereby augmenting the significance of assessing BA [6]. From a legal standpoint, when identification documents of children or adolescents are lacking, estimation of physical maturation is used as an approximation to assess unknown CA [7]. X-ray images, magnetic resonance images (MRI), and computed tomography (CT) are most commonly used for the analysis of bone maturity and age [8]. Recently, ultrasound (US) has been applied to estimate BA aiming to reduce the risks associated with X-ray exposure [9]. In a practical sense, traditional BA assessment methods require professional physicians to evaluate the X-ray images, MRI, CT, or US images, which are time-consuming and prone to subjective variation owing to inter- or intra-observer variability.

Classical methods for determining BA rely on comparing morphological changes to reference atlases of left hand-wrist radiographs, notably the Greulich–Pyle (GP) [10] and the Tanner–Whitehouse (TW) [11,12,13] methods, as well as panoramic dental X-rays like Demirjian. Demirjian is the leading dental method, evaluating seven mandibular teeth using an eight-stage development system [14,15,16]. Despite their effectiveness, BA assessment remains complex, influenced by non-modifiable factors such as genetics and modifiable factors like diet and environment [17]. Variations in skeletal maturity markers across ethnic groups highlight the need for new radiographic age determination methods that consider these racial differences [18]. Overall, while current techniques provide essential insights into skeletal development, their limitations indicate the necessity for refined approaches tailored to diverse populations and individual circumstances.

**Figure 1:**
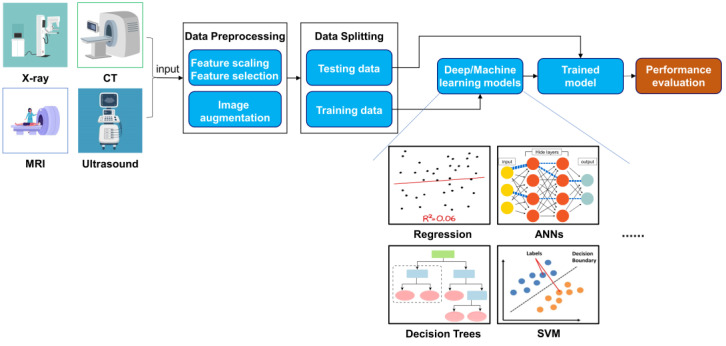
Typical flowchart for AI-based BA assessment.

The limitations of traditional BA assessment methods drive the demand for automated solutions. In 1989, the first automated tool, HANDX [19], emerges as a histogram model that utilizes preprocessing, segmentation, and measurement, marking an early attempt at automation without deep learning (DL) or machine learning (ML) techniques. Recently, researchers have focused on developing reliable automated BA determination tools, leveraging artificial intelligence (AI), which has seen significant advancements in medicine [20]. AI encompasses intelligent machine design that mimics human thought processes. Machine learning (ML) is a subset of AI that utilizes large amounts of structured and unstructured data to create algorithms for accurate predictions. Traditional ML algorithms include regression-based algorithms [21], artificial neural networks (ANNs) [22], support vector machines (SVM) [23], Bayesian networks [24], decision trees (DTs) [25], and K-nearest neighbors (KNN) [26]. DL, a further subset of ML, involves self-training machines using extensive data and complex algorithms to extract information and analyze patterns through multiple processing layers. This technology finds applications across medical research areas, including genomics, diagnosis, and prognosis, aiming to identify patterns in data to enhance decision-making in healthcare [27]. By integrating AI and its subsets, the potential for more precise and efficient BA assessments is significantly increased, addressing the shortcomings of traditional methods [28,29]. The typical process for AI-based BA assessments is presented in Figure 1.

Many recent reviews overview existing BA assessment methods and their clinical applications [29,30,31,32,33,34,35], yet few compare AI-based approaches across various imaging technologies. This article aims to detail the advancements in BA estimation using different medical imaging techniques, particularly focusing on recent ML methods. It highlights the benefits and limitations of each approach and provides a historical context for automated BA assessments. Additionally, the study presents a literature review on AI-based BA assessment across diverse imaging modalities, offering insights into the progress and implications of these technologies in clinical practice. Moreover, despite the progress made in BA assessment using various imaging techniques and AI, there are still significant gaps in the literature. For instance, there is a lack of studies evaluating the performance of AI models in diverse populations, particularly in non-Caucasian groups. Furthermore, there is an opportunity to improve the generalizability and robustness of AI models by incorporating larger and more diverse datasets.

## 2. Benchmark Datasets

Several benchmark datasets have been utilized in AI-based BA assessment studies. The Radiological Society of North America (RSNA) Pediatric Bone Age Challenge 2017 dataset [36], for example, was developed to evaluate the performance of computer algorithms in estimating BA from pediatric hand radiographs. This dataset comprises a substantial number of hand images from children of varying ages. Another notable dataset is the Digital Hand Atlas (DHA) developed by the University of Southern California (USC), comprising 1,400 left-hand and wrist radiographs from children aged 0 to 18 years, representing diverse ethnicities and genders [37]. The DHA is particularly valuable for training and evaluating AI models due to its comprehensive representation of bone development across different age groups and demographic backgrounds. Additionally, the BoneXpert dataset is a commercial AI-based BA assessment system [38]. Although details of this proprietary dataset are not publicly available, it serves as a benchmark for evaluating the BoneXpert system’s performance. Furthermore, many studies leverage local medical image databases from hospitals or universities, which include hand and wrist radiographs, MRI, or other imaging modalities relevant to BA assessment. Beyond the RSNA dataset, other public databases also contain imaging data pertinent to BA assessment, although they may lack the standardization or specificity of the RSNA dataset. These datasets are critical for the development of AI models, providing essential data for training, validation, and performance benchmarking. Researchers frequently compare their models’ performance against these benchmark datasets to ensure the algorithms are generalizable and accurate across diverse populations and imaging conditions.

## 3. BA Assessment Using X-Ray Images

### 3.1. Traditional Methods Using X-Ray Images for BA Assessment

The most commonly employed methods for assessing BA—the GP and TW methods—are based on hand and wrist radiographs. The GP method, introduced in 1959 by William Walter Greulich and Sarah Idell Pyle, utilizes an atlas of reference images derived from hand radiographs of Caucasian children in Ohio, collected from 1931 to 1942 [10]. BA analysis involves comparing an individual’s hand radiograph with the reference images in the atlas [2]. Approximately 76% of radiologists utilize the GP method due to its speed and simplicity; however, it suffers from significant inter-observer and intra-observer variability. Recent studies indicate that secondary sex characteristics in boys and girls in the United States emerge earlier than they did several decades ago, complicating accurate BA assessments in contemporary children using the GP method [39]. While the GP method is deemed applicable and reliable for children in Australia [40] and the Middle East [41], discrepancies between calculated BA and chronological age have been noted when applying this method to Asian children [42].

In contrast, the TW method and its variants, such as TW2 and TW3, focus on the analysis of specific bones rather than the entire hand as in the GP method [43]. Developed from data collected between 1950 and 1960 in the United Kingdom, the TW2 method evaluates maturity scores for the radius, ulna, carpals, and 13 short bones of the hand [44]. These bones are categorized into stages ranging from A to I, resulting in a total score that is then converted into BA. The method was updated in 2001 to the Tanner–Whitehouse 3 (TW3) method, which refined the relationship between total bone maturity scores and BA based on secular trends [12]. Although TW methods are less commonly used due to their time-consuming nature compared to the GP method, they offer greater accuracy and effectiveness. Furthermore, their modular structure makes them suitable for automated BA assessment [45,46].

The Gilsanz–Ratibin (GR) method, developed by Vicente Gilsanz and Osman Ratibin in 2005 [47], provides standardized images representing various stages of skeletal maturity across different ages and sexes. By thoroughly analyzing the size, shape, morphology, and density of ossification centers in hand radiographs of healthy children, the GR method generates images that encapsulate typical developmental characteristics for each ossification center. The images produced by the GR method are of higher precision and quality than those from the GP method. Additionally, GR method standards are spaced at regular six-month intervals from ages 2 to 6 and yearly intervals from ages 7 to 17. Although there is a high degree of agreement between BA assessments using the GP and GR methods among pediatric endocrinologists, the GR atlas has a higher number of outliers. Nevertheless, it serves as a viable replacement for the older GP atlas [1]. Table 1 summarizes the advantages and disadvantages of these traditional approaches to BA estimation.

### 3.2. AI-Based BA Assessment Systems Using X-Ray Images

#### 3.2.1. BA Assessment Based on Hand Radiograph Analysis Using AI Technique

In 2019, Dallora et al. [35] reviewed ML-based automated BA assessment solutions, summarizing their systematic literature and noting that most studies aimed at developing automatic BA assessment systems have focused on hand and wrist radiographs. These X-ray images provide an ideal dataset for training ML models, as they consist of single images of the left hand and wrist with relatively standardized findings. The first semi-automated tool, HANDX, was introduced in 1989, based on left-hand and wrist X-rays. In 1995, Gross et al. developed a neural network for calculating BA using measurements from hand X-rays [32]. Table 2 summarizes recent advancements in AI-based age assessment focused on the hand region.

One of the few commercialized AI-based BA assessment systems, BoneXpert, was introduced in 2008 [38]. This system employs feature extraction techniques to analyze left-hand radiographs of 13 bones, including the radius, ulna, and 11 short bones, to calculate BA. When a radiograph is submitted, BoneXpert uses an active appearance model that has learned the shape and density distribution of each bone based on convolutional neural networks (CNNs) algorithms. The BA is determined based on the GP or TW method. Validated in Caucasian children with short stature and precocious puberty, BoneXpert shows effectiveness across various ethnic groups, particularly after calibration for Japanese children [68]. Zhang et al. [48,57] demonstrate the system’s reliability for BA assessment in Chinese children. In 2018, Lepe et al. [51] investigate the correlation between traditional radiological BA assessment using the GP method and BoneXpert in Brazilian children, finding a strong correlation coefficient of 0.91 to 0.93. Bland–Altman analysis reveals an average difference of 0.19 years (CI: 0.13 to 0.25) between manual assessments and those made with BoneXpert, confirming its accuracy and reliability in diverse populations.

Table 2 outlines the algorithms employed for hand region radiograph analysis for BA estimation, including regression-based methods, CNNs, and SVMs. The majority of studies utilized CNN algorithms. CNNs are a specific type of complex ANNs inspired by the biological neural networks of the human brain, enabling multifactorial analyses. Unlike purely statistical models, ANNs learn from the computational properties of neural ensembles. They consist of a multi-layer structure with nodes connected by weighted edges, forming an input layer, one or more hidden layers, and an output layer. The network is trained by incrementally adjusting the weights based on known input and output values until the output aligns closely with the known output [22]. CNNs have significantly advanced computer vision tasks, allowing for image interpretation, such as localization, segmentation, or detection of specific elements [69]. Systems like BoneXpert leverage CNN methodologies to achieve low mean absolute error (MAE) and root mean square error (RMSE) across diverse populations, demonstrating superior generalizability, particularly in ethnic inclusivity. However, CNN-based BA assessment faces challenges. Training requires large, diverse datasets to avoid overfitting and ensure generalizability, but such datasets remain limited. The computational intensity of CNNs may hinder their adoption in resource-constrained clinical settings. Additionally, the “black box” nature of CNNs reduces interpretability, complicating clinical decision-making. Variability in datasets and performance metrics across studies further complicates direct comparisons, while inadequate reporting on ethnicity and socioeconomic factors raises concerns about bias. Future advancements should prioritize increasing dataset diversity, enhancing model interpretability, and addressing computational barriers. These improvements are essential for equitable and practical deployment of AI-driven BA assessment tools in diverse clinical settings.

Substantial datasets are vital for developing reliable AI approaches. As indicated in Table 2, approximately half of the studies utilized local medical image databases (e.g., hospital or university databases), while nearly half employed public databases such as RSNA. Some studies combined both local and public databases. The origins of the data are detailed in Table 2, with subjects from diverse countries including Europe, America, Asia, and Australia. However, few studies provided comprehensive insights regarding the origin or ethnicity of the subjects, which are crucial factors influencing BA assessment. Additionally, socioeconomic elements, which are vital in the context of any study, were largely overlooked. Tajmir et al. [53] demonstrated similar BA estimation performance across different ethnicities, providing evidence of generalizability. Most studies focused on samples under 18 years of age or bordering this age, with one exception [49]. This focus is justified by legal classifications in many countries, where individuals under 18 are considered minors. Both boys and girls were included in most studies, except for five that did not categorize the samples [52,53,56,58,61,62].

While most studies concentrated on healthy individuals for automated BA assessment, few validated these systems for children with abnormalities. Rassmann et al. [56] presented a DL approach, named Deeplasia, specifically designed for BA assessment in patients with skeletal dysplasia. The mean absolute differences (MAD) for the Deeplasia model were 3.87 months for the normal test set and 5.84 months for the dysplastic set. The performance of models is assessed using various metrics, complicating comparisons between studies. Additionally, the diverse characteristics of the datasets—such as origin, sample size, and age range—further hinder performance comparisons. The results for the performance of the employed techniques are summarized in Table 2, with MAD values ranging from 3.8 to 7.5 months in BA assessments using AI technology.

#### 3.2.2. BA Assessment Based on Clavicle or Tooth Radiograph Analysis Using AI Techniques

Traditionally, hand-wrist radiographs have been widely utilized to determine the growth and developmental stages of individuals. Additionally, BA can be assessed through cervical vertebra maturation stages. Lamparski was the first to observe that cervical vertebrae could serve as indicators of growth and development [70]. This technique was refined by Hassel and Farman [71], and later by Baccetti et al. [72] in 2002 and 2005, to enhance the accuracy of cervical maturation assessment. They identified six stages of cervical maturation based on morphological changes in the C2, C3, and C4 vertebrae using a single lateral cephalogram, regardless of the patient’s gender. Over the past two decades, numerous studies have explored the use of lateral cephalometric X-rays to assess skeletal maturity by examining changes in the shapes of these cervical vertebrae.

Table 3 summarizes the main characteristics of recent studies that have employed clavicle or tooth radiograph analysis for BA estimation. In eight studies, the Cervical Vertebral Maturation Index (CVMI) was utilized to estimate BA [73,74,75,76,77,78,79,80], with six applying the Baccetti and Franchi technique, one adopting the Hassel and Farman approach [73,75,76,77,78,79,80], and another determining the CVMI stage from the Skeletal Maturation Index (SMI) developed by Fishman [74]. Automated models demonstrated effectiveness comparable to manual methods for BA estimation. The highest accuracy was observed at the CS1 (first cervical vertebra maturation stage) [75,76] and CS6 stages [79,80], while the lowest accuracy was noted at the CS3 stage [79,80]. Among various ML models, ANNs and DTs exhibited the highest accuracy in BA estimation, whereas KNN and Logistic Regression (LR) showed the least accuracy [75,77].

Dental maturity assessment offers an alternative method for BA evaluation, as tooth mineralization is less influenced by nutritional or hormonal factors compared to the skeletal system. Zabrowicz et al. [81] develop deep neural network models, achieving mean age differences of 2.34 to 4.61 months and RMSE values between 5.58 and 7.49 months. The correlation coefficient (R^2^) for these models ranges from 0.92 to 0.96. Despite the potential applications of AI in BA assessment based on clavicle or tooth radiographs, the limited number of reported trials suggests that the availability of reliable commercial software remains a distant prospect.

**Table 3:** Main characteristics of studies based on clavicle or tooth radiograph analysis for AI-based BA estimation.

	Author and Year	Data’s Origin	Sample Size	Regions of Interest	Age Range	M/F	Method	Algorithm Used	Performance
1	Bapista et al. (2012) [73]	Brazil	188	clavicle	Not mentioned	69/119	Baccetti et al. [72]	Naive Bayes algorithm	κ 0.992; accuracy: 90.42%
2	Santiago et al. (2014) [74]	Brazil	236	clavicle	Not mentioned	116/120	SMI by Fishmans [82] and quantitative CVM changes	LR	predictability of 81.4%.
3	Kok et al. (2019) [75]	Turkey	300	clavicle	8–17 years	Not categorized	Hassel and Farman [71]	k-NN, DTs, ANNs, SVM, random forest (RF), LR	Not mentioned
4	Makaremi et al. (2019) [76]	France	1870	clavicle	Not mentioned	Not categorized	Baccetti et al. [72]	CNNs	Accuracy 99.0%
5	Amasya et al. (2020) [77]	Turkey	647	clavicle	10–30 years	304/343	Baccetti et al. [72]	LR, SVM, RF, ANNs, DTs	ANNs: κ = 0.926
6	Kim et al. (2021) [78]	Korea	455	clavicle	6–18 years	227/272	Baccetti et al. [72]	CNNs	Accuracy 62.5%
7	Zhou et al. (2021) [79]	China	1080	clavicle	6–22 years	432/548	Baccetti et al. [72]	CNNs	ME 0.36 ± 0.09 mm
8	Rahimi et al. (2022) [80]	Iran	890	clavicle	Not mentioned	Not categorized	Franchi et al. [83]	DL model	accuracy 82.83%
9	Zaborowicz et al. (2022) [81]	Poland	619	tooth	4–15 years	323/296	Computer Vision	deep neural network models	MAE: 2.34–4.61 months; RMSE 5.58–7.49 months

## 4. BA Assessment Using MRI

### 4.1. Traditional Methods Using MRI for BA Assessment

A significant limitation of the commonly used X-ray approach is exposure to ionizing radiation. In recent years, research on age estimation has increasingly focused on MRI as a non-ionizing alternative. MRI technology offers superior contrast resolution, allowing for a more precise assessment of growth plates, particularly with 3.0-T MRI [84]. Terada et al. [85] compared MRI to traditional radiographic methods, utilizing a novel open compact MRI system with a permanent magnet designed for scanning children’s hands. Despite its noninvasive nature, the MRI scanning process is relatively time-consuming, lasting approximately 2 min and 44 s, which may be challenging for young children due to potential movement during the scan [2]. In 2014, Krämer et al. [86] reported a method for BA estimation based on the ossification stage of the distal femoral epiphysis using 3T MRI in individuals aged 10 to 30 years, classifying the stages of epiphyseal fusion into five categories.

### 4.2. AI-Based BA Assessment Systems Using MRI

Table 4 presents nine studies that utilize AI approaches for BA assessment via MRI [87,88,89,90,91,92,93,94,95]. Seven of these studies employ neural networks for analysis [88,89,90,91,92,94,95]. Five focus on knee data [88,89,92,93,94,95], assessing growth plate ossification, while four include hand data [87,90,92,93]. Most datasets come from Caucasian populations, with only two studies obtaining data from China [90,95]. Urschler and Stern [87] developed a regression random forest system trained on 102 males aged 13 to 20 years, achieving a MAD of 0.85 years in age estimation. They later proposed an automatic multifactorial age estimation method, extending the age range to 25 years, using a deep convolutional neural network trained on 322 subjects, yielding a mean absolute prediction error of 1.01 years [91]. Dallora et al. [88] examined 402 subjects aged 14 to 21 years, combining two CNN models: one selected informative MRI, while the other performed age estimation. They reported an MAE of 0.793 years for males and 0.988 years for females. In 2020, they presented models for chronological age estimation and classification of minors versus adults, achieving accuracies of 90% and 84% for male and female subjects, respectively, using MRI examinations of the knee, foot, and wrist [93].

AI-based MRI BA assessment has shown significant advancements in improving the accuracy and efficiency of age estimation. Studies using DL techniques, particularly CNNs, have outperformed traditional methods like manual feature extraction and multi-atlas registration for segmenting growth plates and predicting age. For instance, CNNs have demonstrated exceptional performance in knee MRI segmentation, achieving Dice Similarity Coefficients (DSCs) of up to 98%, surpassing previous benchmarks [89]. This improved segmentation has resulted in reduced prediction errors compared to direct analysis of raw MRI. Notably, segmentation methods have demonstrated robustness to noise, with error rates still smaller than those induced by the noise itself. However, while segmentation significantly improves accuracy, direct age estimation from MRI—without segmentation—remains prone to substantial errors, particularly when unsegmented data are used.

Despite the promising performance, certain limitations still hinder the widespread application of AI-based MRI BA assessment. One key challenge is the training time and dataset limitations, with smaller and non-uniform datasets complicating model generalization. Furthermore, AI models like CNNs may struggle with population biases, such as those related to age, sex, and ethnicity, which could affect the reliability of predictions, particularly when data are not sufficiently diverse. Additionally, DL architectures, while powerful, require substantial computational resources, making their application in real-world clinical settings more challenging. Alternative methods, such as Mixed-Scale Dense Networks [89], may offer more flexibility and improved performance, particularly in handling varying feature scales and enhancing model robustness. The comparison of AI models to traditional methods further highlights the advantages of CNNs over older algorithms such as DTs, RF, and SVMs. For example, models like AgeNet2D [94], which use 2D CNNs on segmented knee MRIs, have outperformed non-CNN methods in age regression tasks, achieving MAEs as low as 0.5 years. However, 3D CNNs [89,94], despite offering enhanced performance by utilizing spatial information across MRI volumes, still lag behind in some cases, particularly due to issues like underfitting and insufficient dataset sizes. This points to the ongoing need for larger, more diverse datasets to train AI models effectively.

## 5. BA Assessment Using CT

### 5.1. Traditional Methods Using CT for BA Assessment

CT is increasingly utilized in forensic anthropology, providing researchers with a substantial dataset for age estimation. CT scans offer clear visualization of bone structures, enabling even less experienced researchers to analyze bones effectively. In 1998, Kreitner and colleagues [96] first applied CT scanning for age estimation by assessing the medial clavicular epiphysis (MCE). Later, in 2004, Schmeling and colleagues [97] developed a five-stage method that begins with a non-ossified ossification center and concludes with complete ossification and union with the metaphysis. This staging system correlates well with age; however, stages 2 (non-union ossified epiphysis) and 3 (partially unioned growth plate) encompass a wide age range. To enhance estimation accuracy, Kellinghaus and colleagues [98] refined this method by introducing a sub-stage system that further divides stages 2 and 3 into sub-stages a, b, and c, based on the size of the ossified epiphysis and the extent of the union in growth plates. Currently, CT scans are predominantly used to evaluate the MCE for forensic age estimation.

### 5.2. AI-Based BA Assessment Systems Using CT

Table 5 summarizes the main characteristics of recent studies utilizing CT analysis for BA estimation. These studies employed various regions of interest (ROIs), including the [99], lumbar [100], clavicle [101] and vertebrae [102]. Notably, the age ranges for BA assessment using CT were significantly older than those for radiographic and MRI methods. Automated models have proven effective in using CT for BA estimation. Qiu et al. [101] developed ML and DL models based on medial clavicle CT images, evaluating their performance on both normal and variant clavicles. They found that the SVM achieved the best MAE of 1.73 years, followed by commonly used CNNs with MAEs ranging from 1.77 to 1.93 years, a linear model at 1.94 years, and the hybrid neural network CoAt Net at 2.01 years. Among the DL models, SE Net 18 performed best, yielding results similar to the SVM in the normal test set and achieving an MAE of 2.08 years in the external variant test.

Studies on AI-based CT bone age assessment show that texture analysis combined with ML techniques offers a promising alternative to traditional methods like hand radiographs. Using vertebral bone structure provides a broader and more accurate range for age estimation, with texture features from cancellous bone outperforming DL models in some cases. DL models, such as DenseNet, ResNet, and MobileNet, are limited by factors like insufficient image quality, smaller ROI sizes, and improper scaling [102]. While DL models excel in age and sex determination, they often suffer from overfitting and high sensitivity to data variability, especially with small or unbalanced datasets. In contrast, texture analysis improves accuracy by exploiting structural changes across larger bone regions, even with inconsistent or low-resolution images. However, DL methods remain superior for age estimation beyond adolescence when specialized features like MCE stages are needed. ML models, such as SVM, also handle ordinal data well [101]. Despite progress, challenges remain in image quality, dataset diversity, and model interpretability. Future research should explore hybrid models combining texture analysis and DL, and expand datasets to enhance generalizability across populations and imaging protocols.

## 6. BA Assessment Using US

### 6.1. Traditional Methods Using US for BA Assessment

Several reports have highlighted the ultrasonographic evaluation of BA using a device called BonAge [1,103]. This US device consists of a probe connected to a primary unit that performs BA calculations. The method employs two transducers: one emits ultrasonic waves at a frequency of 750 kHz toward the epiphyses of the distal ends of the ulna and radius, while the other acts as the receiver. The assessment procedure lasts approximately five minutes, during which eleven measurement cycles are conducted to ensure precise outcomes. BA is calculated by integrating the individual’s demographic data with the US findings. Although the use of US for BA estimation is still in its early stages and requires further development, preliminary studies comparing US results with the GP atlas standards have shown promising outcomes [103]. Khan et al. [104] noted that the BonAge device tends to overestimate delayed BA and underestimate advanced BA compared to both the GP and TW3 methods, concluding that BonAge should not be considered a valid substitute for determining radiographic BA. Additional studies assessing BA through ultrasonography are needed, particularly involving larger populations, diverse ethnic groups, and children with growth disorders.

### 6.2. AI-Based BA Assessment Systems Using US

Research on AI applications in ultrasonic biological age assessment is currently limited, as shown in Table 6. In 2022, Yan et al. [105] proposed a weakly supervised interpretable framework called USB-Net, utilizing ultrasonic pelvic images for BA assessment. They created a dataset comprising 1,644 ultrasonic hip joint images, and the model achieved an MAE of 16.24 days on this dataset. Faierstein et al. [106] trained a convolutional neural network algorithm to estimate age and predict sex using standard transthoracic echocardiography, evaluating its prognostic implications. The algorithm was trained on 76,342 patients, validated in 22,825 patients, and tested on 20,960 patients. It was externally validated using data from a different hospital (n = 556). The MAE in age estimation was 4.9 years, with a Pearson correlation coefficient of 0.922, and the accuracy of sex prediction was 96.1%. Although the use of US for BA assessment is still in its early stages, it offers several advantages over traditional imaging modalities, such as its portability, safety, and real-time visualization capabilities. AI-based US methods for BA assessment have shown promise as radiation-free, non-invasive alternatives to traditional radiographic techniques. Among the various ML models applied, DL algorithms, particularly CNNs and hybrid architectures such as CNN-transformers, have achieved considerable success in processing US images to estimate BA. These models have demonstrated strong performance by focusing on critical anatomical regions, such as the ossification center or the acetabulum, which are indicative of skeletal maturity. For instance, the weakly supervised USB-Net, a two-stage network, has proven to be an efficient and interpretable approach, achieving an MAE of 16.24 days on the USBAA dataset, thus highlighting US as a viable modality for BA assessment [105]. Additionally, techniques such as the ossification ratio (OR) and scoring systems, like the ultrasonic skeletal maturity score (SMS), have demonstrated high accuracy in predicting bone age, with sensitivity and specificity exceeding 90% in various populations [107].

However, despite these advancements, AI-based US BA assessment methods are not without limitations. While DL models show promise, the reliance on large annotated datasets remains a significant challenge, particularly for US images, which require precise segmentation and feature extraction. Methods such as ROI-based approaches have enhanced accuracy by focusing on specific anatomical features, but these models require extensive manual annotations, limiting their scalability [105]. Furthermore, AI models trained on small or homogeneous datasets may suffer from limited generalizability across diverse populations, which is crucial for ensuring widespread clinical applicability. For example, although the USTW3 method has been effective in Chinese children [9], there is limited evidence supporting its application across other ethnic groups. Additionally, US’s dependence on operator skill and equipment quality can introduce variability in the results, affecting the consistency and reproducibility of bone age assessments.

## 7. Discussion

X-ray imaging remains the gold standard for BA assessment due to its availability, low cost, and established reference methods such as the GP and TW methods. X-rays produce clear images of skeletal maturation, making them suitable for regression-based algorithms and CNNs, which excel at feature extraction from 2D images [108]. CNNs effectively classify and predict BA by learning features directly from pixel data, minimizing the need for manual feature engineering. MRI, with its superior soft tissue contrast, is valuable for complex cases of growth anomalies, utilizing DL algorithms such as ANNs and SVMs for nuanced interpretations of skeletal patterns [109]. CT imaging offers detailed views useful for assessing skeletal dysplasias, enhancing the performance of algorithms like Bayesian networks, which can model uncertainty in data and incorporate prior knowledge of bone growth [110]. Although less conventional, US is gaining popularity due to its portability and safety, allowing real-time visualization of growth plates. In this context, k-NN actually can be classified based on similarity [111]; however, their effectiveness depends on distance metrics and dataset size [112], emphasizing the need for diverse training data. Overall, integrating imaging technologies with AI enhances the accuracy and efficiency of BA assessments across various methodologies.

The choice of DL algorithms for BA assessment depends on the dataset’s characteristics and the challenges specific to each imaging technique. Regression-based algorithms are effective for continuous outputs, such as exact BA, while classification methods, such as DTs, provide intuitive interpretations of age brackets that are valuable in clinical settings. Hybridizing algorithms can improve the robustness of BA predictions; for example, combining CNNs with DTs or Bayesian networks can extract complex features while offering a probabilistic decision-making framework. The utilization of ML technologies is essential for facilitating automatic BA assessment solutions. This is particularly evident in the application of CNNs in computer vision tasks, which differ from regression-based methods that require some level of human intervention. Compared to other medical imaging techniques, studies on BA assessment have predominantly focused on methodologies involving hand and wrist radiographs. However, using radiative methods without therapeutic purposes raises ethical concerns. While CT, MRI, US, and other imaging techniques serve as supplements to X-ray imaging in BA assessment, the limited number of attempts in these areas means that the commercialization of reliable software remains a distant goal.

## 8. Challenges and Perspectives

**Figure 2:**
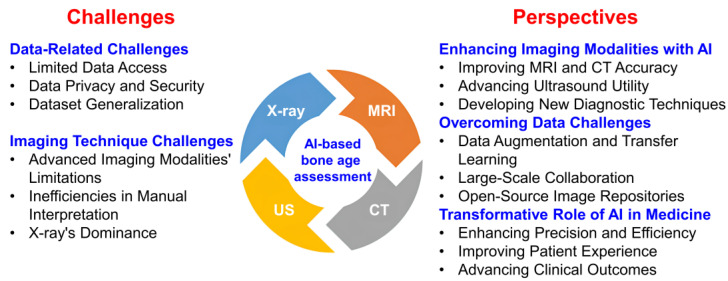
Challenges and perspectives for AI-based BA assessment across various medical imaging techniques.

Despite advancements, challenges persist in AI-based BA assessment, including variability in imaging techniques, differing growth patterns, and the need for large, well-annotated datasets (Figure 2). Addressing these issues requires overcoming limitations in data access, generalization, and data privacy. Access to extensive, diverse datasets is essential for training and validating AI models, with efforts like open-source image repositories and data-sharing initiatives helping to mitigate this challenge. However, most studies lack detailed information on sample origins, neglecting crucial factors like ethnicity and socioeconomic status. These oversights hinder AI model performance across different populations. Additionally, variations in imaging equipment and patient demographics limit the generalizability of models to diverse clinical settings. Data privacy and security also remain significant concerns, as medical images contain sensitive personal information. Ensuring compliance with regulations and safeguarding data during storage and sharing are essential for maintaining the integrity of AI systems. Innovations in data augmentation, transfer learning, and large-scale collaboration can help address these obstacles, advancing the accuracy and interpretability of AI-assisted BA assessments.

X-ray imaging remains the cornerstone of BA assessment not only because of its simplicity, accessibility, and reliability in clinical practice but also because of its long history of extensive data accumulation and clinical expertise. Before the integration of AI, advanced imaging modalities such as MRI, CT, and US faced significant challenges, including high costs, complexity, and limited availability. MRI posed risks such as contraindications and prolonged examination times, while CT raised concerns about radiation exposure. The US lacked standardized scoring methods, limiting its clinical use. Traditional manual interpretation of these images further introduced inefficiencies and human error, hindering the routine adoption of these advanced technologies in BA assessment. The introduction of AI has addressed these challenges by enhancing imaging quality, streamlining diagnostic workflows, and enabling precise analysis of anatomical features like growth plates. Moreover, AI ensures consistent and objective analysis, thereby minimizing variability in assessments. It has also created significant opportunities to rapidly establish databases and models for these advanced imaging modalities, bypassing the need for prolonged data accumulation.

AI integration with MRI, CT, and US improves the accuracy and reliability of these modalities, especially for pediatric patients, as MRI and US are radiation-free and suitable for repeated evaluations (Figure 2). By combining MRI’s soft tissue details with CT’s structural insights, AI offers a more comprehensive evaluation of skeletal maturity. Moreover, AI has facilitated the development of new diagnostic techniques, including DP models and automated evaluation protocols. AI also enhances the clinical utility of US, making it a non-invasive, cost-effective option for BA assessment. These advancements expand the application of US while reducing radiation exposure, particularly important in pediatric care. In summary, AI is transforming imaging technologies, complementing traditional X-ray methods, and improving clinical outcomes. AI’s transformative role enhances precision, efficiency, and safety, ultimately improving patient experience and outcomes in modern medicine.

## Figures and Tables

**Table 1:** Advantages and disadvantages of traditional approaches for BA estimation.

Traditional Method	Procedure	Advantages	Disadvantages
Greulich–Pyle (GP)	comparison with reference images contained in the Atlas	wide availability longtime experience	affected by ethnicity and generation differences affected by scarce inter-observer and intra-observer
Gilsanz–Ratibin (GR)	comparison with reference images contained in the Digital Atlas	wide availability longtime experience high-quality digital images	affected by ethnicity and generation differences affected by inter-observer and intra-observer
Tanner–Whitehouse (TW)	scoring the level of maturity of specific regions of interest based on the reference scale	more precise than GP and GR methods higher accuracy and reproducibility	time-consuming affected by ethnicity and generation differences

**Table 2:** Main characteristics of studies based on hand region radiograph analysis for AI-based BA estimation.

	Author and Year	Data’s Origin	Database	Sample Size	Regions of Interest	Age Range	M/F	Algorithm Used	Performance
1	Zhang et al. (2016) [48]	China	Local dataset	397	hand and wrist	2–14 years	229/168	BoneXpert	Not mentioned
2	Maggio et al. (2016) [49]	Australia	Local dataset	360	Hand, Wrist	0–24 years	180/180	Polynomial Regression	SEE: 1.31 (male) and 2.37 years (female)
3	Lee et al. (2017) [50]	The USA	Local dataset	8325	hand and wrist	5–18 years	4278/4047	CNNs	Accuracy: 61.40% (male) 57.32% (female)
4	Lepe et al. (2018) [51]	Chile	Local dataset	1493	hand and wrist	<16 years	471/922	BoneXpert	Not mentioned
5	Ren et al., (2018) [52]	China	RSNA dataset and local dataset	12,480	hand and wrist	0-18 years	Not categorized	CNNs	MAE 0.43 y
6	Tajmir et al. (2019) [53]	The USA	Local dataset	280	hand and wrist	5–18 years	Not categorized	CNNs	RMSE:0.60 y
7	Lee et al. (2021) [54]	The USA	RSNA dataset	2800	hand and wrist	2–18 years	51/51	CNNs	MAD: 0.74 y
8	Liu et al. (2024) [55]	China	RSNA dataset, RHPE dataset and a local dataset	21,951	hand and wrist	0–18 years	11,249/10,702	DL model	MAD: RSNA: 0.34 y; RHPE: 0.52 y; local dataset: 0.47 y
9	Rassmann et al. (2024) [56]	German	RSNA dataset and dysplastic set	14,804	hand and wrist	0–18 years	Not categorized	CNNs	MAD: RSNA: 0.32 y; dysplastic set: 0.49 y
10	Zhang et al. (2013) [57]	China	Local dataset	6026	hand	2–20 years	2883/3143	BoneXpert	Not mentioned
11	Kashif et al. (2016) [58]	The USA	DHA of the USC	1101	hand	0–18 years	Not categorized	SVM	MAE: 0.61 y
12	Kim et al. (2017) [59]	Korea	Local dataset	200	hand	3–17 years	87/113	DL model	Not mentioned
13	Larson et al. (2018) [60]	The USA	Local dataset	14,036	hand	8–20 years	7606/6430	CNNs	RMS: 0.63 y; MAD:6 months
14	Zhao et al. (2018) [61]	The USA	RSNA dataset	12611	hand	0–19 years	Not categorized	CNNs	MAE:0.64 y
15	Umer et al. (2023) [62]	The USA	RSNA dataset	12,600	hand	0-18 years	Not categorized	CNNs	97% accuracy
16	Beheshtian et al. (2023) [63]	The USA	RSNA dataset and DHA of the USC	15,238	hand	0-18 years	8220/7018	DL model	MAD: RSNA 0.57 y; DHA: 0.58 y
17	Suh et al. (2023) [64]	Korea	Local hospital	1678	hand	3-18 years	877/801	CNNs	MAE: 0.59 y RMSE: 0.55 y,
18	Santomartino et al. (2024) [65]	The USA	RSNA dataset and DHA of the USC	2627	hand	0-18 years	1387/1240	DL model	MAD: RSNA: 0.57 y, DHA: 0.58 y
19	Haghnegahdar et al. (2024) [66]	Asian	DHA of the USC	304	hand	1-20 years	149/155	multilayer perception neural network	MAD: 0.65 y (male); 0.83 y (female)
20	Grafe et al. (2024) [67]	German	Local dataset (Caucasian in 294, 96%)	306	hand	1–18 years	153/153	IB Lab PANDA	MAD: 0.57 y

**Table 4:** Main characteristics of studies using MRI for AI-based BA estimation.

	Author and Year	Data’s Origin	Sample Size	Regions of Interest	Age Range	M/F	Algorithm Used	Performance
1	Urschler et al. (2015) [87]	Austria	102	hand and wrist	13–20 years	102/0	Regression RF	MAD: 0.85 y
2	Dallora et al. (2019) [88]	Sweden	402	knee	14–21 years	221/181	CNNs	MAE: 0.793–0.988 y; Accuracy 95–98.1%
3	Prove et al. (2019) [89]	Germany	150	knee	14–20 years	150/0	CNNs	MAE: 0.48 ± 0.32 years
4	Tang et al. (2019) [90]	China	79	hand and wrist	12–17 years	39/40	ANNs	MAD: 0.13 y (male) and 0.08 y (female)
5	Stern et al. (2019) [91]	Austria	322	hand, clavicle, and tooth	13–25 years	Not categorized	CNNs	MAE: 1.01 ± 0.74 y
6	Stern et al. (2019) [92]	Austria	328	hand	13–25 years	Not categorized	RF, DCNN	MAE: 0.37 ± 0.51 y
7	Dallora et al. (2020) [93]	Sweden	938	knee, foot, and wrist	14–21 years	465/473	DT, RF, multilayer perceptron, SVM, naïve Bayes, and KNNs.	Accuracy: 90% (male) and 84% (female) MAE: 0.95 y (male), 1.24 y (female)
8	Mauer et al. (2021) [94]	Germany	589	knee	13–21 years	376/213	CNNs	MAE: 0.67 ± 0.49 y; accuracy: 90.9%
9	Fan et al. (2024) [95]	China	598	knee	10–29 years	353/245	CNNs	MAE: 1.32 ± 1.01 y (10–25 years)

**Table 5:** Main characteristics of studies based on CT for AI-based BA estimation.

	Author and Year	Sample Size	Regions of Interest	Age Range	M/F	Algorithm Used	Performance
1	Kerber et al. (2023) [99]	1653	thorax and abdomen	20–85 years	1120/533	DL (ResNet- 18)	MAE: 5.76 ± 5.17 y
2	Levi et al. (2023) [100]	233	lumbar	mean age 59.8 years (male) 60.6 years (female)	126/107	SVM, LR	MAD 7.232 y
3	Qiu et al. (2023) [101]	1049	clavicle	14–29.99 years	500/549	SVM, CNNs, linear model, hybrid neural network CoAt Net, SE Net 18	MAE: SVM: 1.73 y, CNNs: 1.77–1.93 y, linear model: 1.94 y, hybrid neural network CoAt Net: 2.01 y, SE Net 18: 2.08 y
4	Nurzynska et al. (2024) [102]	166	vertebrae	21–84 years	95/71	MLP based regression	MAE: 3.14

**Table 6:** Main characteristics of studies based on US for AI-based BA estimation.

	Author and Year	Sample Size	Regions of Interest	Age Range	M/F	Algorithm Used	Performance
1	Yan et al. (2022) [105]	1644	pelvis	21–223 days	694/950	weakly supervised method	MAE: 16.24 days
2	Faierstein et al. (2024) [106]	120127	heart	>18 years	Not categorized	CNNs	MAE: 4.9 y
